# *Moringa oleifera* Seed Extract Alleviates Scopolamine-Induced Learning and Memory Impairment in Mice

**DOI:** 10.3389/fphar.2018.00389

**Published:** 2018-04-24

**Authors:** Juan Zhou, Wu-shuang Yang, Da-qin Suo, Ying Li, Lu Peng, Lan-xi Xu, Kai-yue Zeng, Tong Ren, Ying Wang, Yu Zhou, Yun Zhao, Li-chao Yang, Xin Jin

**Affiliations:** ^1^Department of Obstetrics and Gynecology, The First Affiliated Hospital of Xiamen University, Xiamen University, Xiamen, China; ^2^Department of Neurosurgery, Xiamen Hospital of Traditional Chinese Medicine, Xiamen, China; ^3^Xiamen Key Laboratory of Chiral Drugs, Medical College, Xiamen University, Xiamen, China; ^4^Department of Pharmacy, Xiamen Medical College, Xiamen, China

**Keywords:** *Moringa oleifera*, Alzheimer’s disease, scopolamine, acetylcholine, neurogenesis

## Abstract

The extract of *Moringa oleifera* seeds has been shown to possess various pharmacological properties. In the present study, we assessed the neuropharmacological effects of 70% ethanolic *M. oleifera* seed extract (MSE) on cognitive impairment caused by scopolamine injection in mice using the passive avoidance and Morris water maze (MWM) tests. MSE (250 or 500 mg/kg) was administered to mice by oral gavage for 7 or 14 days, and cognitive impairment was induced by intraperitoneal injection of scopolamine (4 mg/kg) for 1 or 6 days. Mice that received scopolamine alone showed impaired learning and memory retention and considerably decreased cholinergic system reactivity and neurogenesis in the hippocampus. MSE pretreatment significantly ameliorated scopolamine-induced cognitive impairment and enhanced cholinergic system reactivity and neurogenesis in the hippocampus. Additionally, the protein expressions of phosphorylated Akt, ERK1/2, and CREB in the hippocampus were significantly decreased by scopolamine, but these decreases were reversed by MSE treatment. These results suggest that MSE-induced ameliorative cognitive effects are mediated by enhancement of the cholinergic neurotransmission system and neurogenesis via activation of the Akt, ERK1/2, and CREB signaling pathways. These findings suggest that MSE could be a potent neuropharmacological drug against amnesia, and its mechanism might be modulation of cholinergic activity via the Akt, ERK1/2, and CREB signaling pathways.

## Introduction

Alzheimer’s disease (AD) is a neurodegenerative disease characterized by deterioration of cognitive and behavior function due to cholinergic nervous system dysfunction (Scarpini et al., 2003). As primarily observed in patients with AD, decreased cholinergic function in the brain can lead to a decline in memory and cognitive function (Hasselmo, 2006). In clinical studies, a number of cholinergic drugs have been approved to treat or ameliorate AD, and they exert therapeutic effects by preventing acetylcholine (ACh) insufficiency and consequently increasing ACh levels in the brain (Drever et al., 2007). In fact, acetylcholinesterase (AChE) inhibitors are the most common class drugs for AD, such as galantamine, rivastigmine, and donepezil, which temporarily enhance the availability of ACh at cholinergic synapses (Lanctot et al., 2009). However, the number of drugs approved for the treatment of AD patients with cognitive impairment is limited due to their side effects, which include pain, nausea and hepatotoxicity (Lahiri et al., 2003). Therefore, an alternative treatment strategy for AD patients is required.

Scopolamine is a tropane alkaloid drug that produces competitive antagonism at muscarinic acetylcholine receptors (mAChRs) by interfering with cholinergic transmission, leading to impairing learning and short-term memory in rodents and humans (Iversen, 1997). Therefore, scopolamine administration is used in animals as an experimental model of the memory deficits and cognitive impairment observed in AD. This dementia model has been used to screen for drugs that have potential therapeutic value in AD-type dementia patients (Bartus et al., 1982). Scopolamine administration not only induces dysregulation of the cholinergic system and memory circuits in the brain but also decreases the expression of cAMP-response element binding protein (CREB) and brain-derived neurotrophic factor (BDNF) in the central nervous system (CNS). BDNF is responsible for synaptic plasticity and memory performance and is coupled to CREB activation. CREB is closely related to hippocampal learning and memory (Tyler et al., 2002). Moreover, hippocampal BDNF and CREB play vital roles in pathological conditions and neurodegenerative diseases such as AD. Therefore, the BDNF/CREB pathway may act as a novel therapeutic target to treat cognitive deficits.

*Moringa oleifera* (*M. oleifera*) is one of the Moringaceae (Abdull Razis et al., 2014). *M. oleifera*, by virtue of its high nutritional as well as ethno-medical values, has gained profound interest both in nutrition and medicinal research (Al-Malki and El Rabey, 2015). The leaves, roots, seeds, bark, fruits, flowers, and stem of *M. oleifera* have been shown to exert various pharmacological effects. Additionally, aqueous and ethanolic *M. oleifera* seed extract (MSE) has been shown to possess various pharmacological and commercial utility, such as metal antidote, anti-oxidant, anti-asthmatic, anti-arthritic, anti-bacterial, anti-tumor, and hepatoprotective effects (Abdull Razis et al., 2014). However, few reports have addressed the benefits of ethanolic MSE in diseases involving brain dysfunction. To elucidate the potential effects of MSE on cognitive function and the cholinergic system, we assessed learning and memory retention using the passive avoidance and Morris water maze (MWM) tests and evaluated cholinergic markers (ACh, AChE, and ChAT) in mice exposed to scopolamine.

## Materials and Methods

### Animals

Male ICR mice (6 weeks old, 25–30 g) were purchased from Beijing Vita River Experimental Animal Co. (Beijing, China) and housed under a 12/12 h dark/light cycle and specific pathogen-free (SPF) conditions. The experimental protocols were approved by the Animal Care and Use Committee of the Medical College of Xiamen University in compliance with the NIH Guide for the Care and Use of Laboratory Animals (NIH Publications No. 80-23).

### Drug Preparation

Dry *M. oleifera* seeds were purchased from LuYan Pharma Co. (Fujian Province, China). Fifty grams of dried *M. oleifera* seed were extracted with 0.5 L of 70% ethanol at 85°C for 2 h, and the suspension was filtered using 300-mesh 50-mm filter paper (Advantec, Toyo Roshi Kaisha, Tokyo, Japan). Filtrate was concentrated in a rotary evaporator and lyophilized. The final yield of the extract was stored at -80°C.

### Drug Administration

Animals were randomly assigned to the following groups of 12 mice each: control (saline), scopolamine (4 mg/kg) plus saline (scopolamine-treated control), scopolamine plus MSE (250 mg/kg), and scopolamine plus MSE (500 mg/kg). MSE and scopolamine were dissolved in sterile saline containing 10% Tween-80. MSE was administered by oral gavage (*p.o*.) once a day for 7 or 14 consecutive days, according to the different treatment conditions listed in **Supplementary Figure S1**, and the mice in the control and scopolamine-treated (*i.p*.) groups were treated with the same volume of vehicle for the same duration. Behavioral tasks were performed 24 h after MSE administration daily for 7 days.

### Step-Down Test

The bottom of the apparatus consisted of a cupreous grid, and a platform made of rubber was placed in the center of the apparatus. The mouse was placed on the apparatus without electric shock for 3 min to adapt to the environment before the trial. The mouse was subsequently placed on the platform, and the cupreous bottom received an intermittent electric shock. The mouse was shocked if it stepped down from the platform to the cupreous bottom; it would then step on the platform again. The number of errors (i.e., number of times the mouse stepped onto the platform and received an electric shock) and latency (i.e., time until the mouse first stepped down onto the cupreous bottom with four paws) was recorded in the training phase. Twenty-four hours after training, the retention task was administered to the mouse: it was placed on the platform, and the latency and the number of errors within 300 s were recorded as measures of learning.

### Step-Through Test

The passive avoidance step-through task was also used to measure associative memory performance. The equipment for this test comprised 2 equal compartments (20 cm × 20 cm × 20 cm) separated by a grid door (5 cm × 5 cm). For the acquisition trial, mice were initially placed in the illuminated compartment, and the door between the two compartments was opened 20 s later. The time taken for a mouse to enter the dark compartment (step-through latency) was recorded. Upon entering the dark compartment, the door was closed, and an electrical foot shock (0.5 mA for 5 s) was delivered through the stainless-steel rods. On the second day, the same procedure was followed. The mice were again placed in the illuminated compartment to test retention. The step-through latency was measured as a measure of retention (Worms et al., 1989). If the mouse did not enter the dark compartment within 300 s, it was assumed that the mouse had remembered the single acquisition trial experience.

### Morris Water Maze Test

The MWM was used to test spatial learning and memory (Morris et al., 1982) and was begun from day 8 to day 14 after MSE treatment. For acquisition trial, every mouse was trained four trials per day from days 9 to 13 after MSE administration. Mice were slightly placed into the water from the wall of the pool. The escape latency of finding the hidden platform was recorded during each acquisition trial. The whole time was 90 s. On the last day (day 14), all mice were subjected to probe test without platform, and were recorded for 90 s. The time spent in the target quadrant and the number crossed the platform position was measured for spatial memory.

### ACh and AChE Estimation by Assay Kits

Mice were anesthetized with chloral hydrate (0.4 ml/kg), and their brains were removed. The hippocampus was taken out and was divided into two pieces. One piece was rapidly frozen in liquid nitrogen and stored at -80°C for subsequent Western blot analysis; the second piece was used to assess ACh and AChE using an assay quantification kit (Nanjing Jiancheng Biological Instrument Company, China). Half of the hippocampus was homogenized with assay buffer (0.1 g/0.9 ml) and centrifuged at 3200 r/min for 10 min; the supernatant was then removed. The supernatant was used for estimating ACh and AChE content according to the instructions for the acetylcholine assay kit.

### Immunohistochemistry and Cell Counting

Mice were anesthetized with chloral hydrate and perfused transcardially with ice-cold saline followed by perfusion with 4% paraformaldehyde 24 h after reperfusion. The brains were removed and post-fixed overnight for 24 h in paraformaldehyde; they were then coronally sectioned (30 μm) using a vibrating microtome (Leica, Wetzlar, Germany). The sections were incubated in PBS containing 0.5% Triton X-100 and 10% normal goat serum for 1 h at room temperature, following by incubation with rat monoclonal anti-BrdU (1:400; Abcam, Cambridge, United Kingdom) at 4°C overnight. After several PBS rinses, sections were incubated with Alexa Fluor 594 donkey anti-rat IgG (1:200; Invitrogen, Carlsbad, CA, United States).

An experimenter (L-cY) coded all slides from the experiments before quantitative analysis. All BrdU-labeled cells in the DG of injured hemisphere were counted in each section by another experimenter (D-qS) blinded to the study coding. The total number of BrdU-labeled cells per section was determined and multiplied by 10 to obtain the total number of cells per DG using fluorescence confocal microscopy (EX61; Olympus, Tokyo, Japan).

### Western Blot Analysis

Brain samples were obtained from the hippocampal tissue of mice 24 h after the MWM test. Hippocampal tissue was homogenized with lysis buffer (50 nM/L NaCl, 1 mM/L EDTA, 1% Triton X-100, 0.5% SDS, 0.5% sodium deoxycholate, and 20 mM/L Tris HCl; pH 7.5) and centrifuged at 15,000 × *g* for 20 min. Protein samples (50 μg) per lane were run on polyacrylamide gel, transferred to a PVDF membrane (Millipore, Billerica), and blocked with 5% milk solution (non-fat dry milk in PBST) for 2 h. The membrane was incubated at 4°C overnight with the following specific antibodies: rabbit polyclonal anti-ChAT (1:1000; Cell Signaling Technology, Boston, MA, United States), phospho-Akt (1:1000; Cell Signaling Technology), Akt (1:1000; Cell Signaling Technology), phospho-CREB (1:1000; Cell Signaling Technology), CREB (1:1000; Cell Signaling Technology), ERK1/2 (1:1000, Cell Signaling Technology), phospho-ERK1/2 (Thr202/Thr204) (1:1000, Cell Signaling Technology), BDNF (1:1000, Cell Signaling Technology), NR1 (1:1000, Cell Signaling Technology), NR2B (1:1000, Cell Signaling Technology), GAP-43 (1:1000, Cell Signaling Technology), and mouse monoclonal anti-β-actin (1:10000; Sigma). After washing with TBST five times, the membranes were then incubated with the corresponding conjugated anti-rabbit IgG (1:10000; Cell Signaling Technology) at room temperature for 1 h. Immunoreactive proteins were quantified using an enhanced chemiluminescence (ECL) kit (Millipore, Billerica), and the relative density of the protein bands was scanned using an LAS 4000 Fujifilm imaging system (Fujifilm, Tokyo, Japan) and analyzed by densitometric evaluation using Quantity-One software (Bio-Rad Hercules, CA, United States).

### Statistical Analysis

All data are expressed as the mean ± SEM. We performed the statistical assay using two-way analysis of variance (ANOVA) and one-way ANOVA. The differences between the groups were analyzed by Bonferroni’s *post hoc* test (Prism 5 for Windows, GraphPad Software, Inc., United States). *P* < 0.05 was considered statistically significant.

## Results

### Effects of MSE on Scopolamine-Induced Memory Impairment in the Step-Down Avoidance Test

**FIGURE 1:**
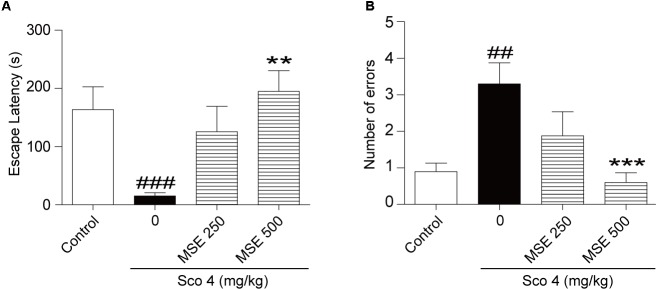
Effects of *Moringa oleifera* seed extract (MSE) on scopolamine-induced memory impairments on the step-down test. MSE (250, or 500 mg/kg, *p.o*.) or the same volume of vehicle (10% Tween 80 solution) was administered to mice by oral gavage for 7 days before the acquisition trial. Memory impairment was induced by scopolamine (4 mg/kg, *i.p*.) 30 min before the acquisition trial. Twenty-four hours after the acquisition trial, a retention trial was conducted for 300 s. Escape latency **(A)** and number of errors **(B)** of mice were detected after training. Data are expressed as the means ± SEM. *N* = 12 for each group. ^##^*P* < 0.01, ^###^*P* < 0.001 vs. control group, ^∗∗^*P* < 0.01, ^∗∗∗^*P* < 0.001 vs. scopolamine+vehicle group.

We assessed the effects of MSE on scopolamine-induced cognitive dysfunction using the step-through passive avoidance task. Compared with control mice, mice treated with scopolamine exhibited reduced escape latencies (**Figure 1A**) and increased errors (**Figure 1B**). In contrast, escape latencies of animals pretreated with 500 mg/kg of MSE for 7 days were significantly longer than those of scopolamine-treated animals (**Figure 1A**), but there was no significant difference between the 250 mg/kg MSE and scopolamine groups (**Figure 1A**). Meanwhile, the number of errors in the 500 mg/kg MSE group was lower than that in the scopolamine group (**Figure 1B**).

### Effects of MSE on Scopolamine-Induced Memory Impairment in the Step-Through Avoidance Test

**FIGURE 2:**
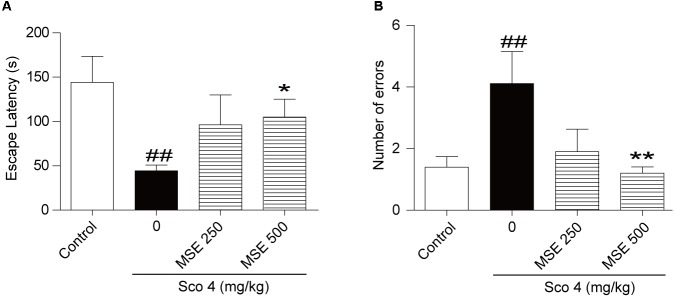
Effects of MSE on scopolamine-induced memory impairments on the step-through test. MSE (250, or 500 mg/kg, *p.o*.), or the same volume of vehicle (10% Tween 80 solution) was administered to mice by oral gavage for 7 days before the acquisition trial. Memory impairment was induced by scopolamine (4 mg/kg, *i.p*.) 30 min before the acquisition trial. Twenty-four hours after the acquisition trial, a retention trial was conducted for 300 s. Escape latency **(A)** and number of errors **(B)** of mice were detected after training. Data are expressed as the means ± SEM. *N* = 12 for each group. ^##^*P* < 0.01 vs. control group, ^∗^*P* < 0.05, ^∗∗^*P* < 0.001 vs. scopolamine+vehicle group.

After the step-down test, we detected the effects of MSE on scopolamine-induced cognitive impairment by the step-through test. As shown in **Figure 2**, escape latencies of animals treated with scopolamine were decreased, and error frequency was increased in comparison with the control group (**Figures 2A,B**). In contrast, the effect of scopolamine on escape latencies and error frequency was reversed by 500 mg/kg of MSE (**Figures 2A,B**; *P* < 0.05, *P* < 0.01, respectively). However, 250 mg/kg of MSE had no effect on scopolamine-induced memory impairment (**Figures 2A,B**).

### Effects of MSE on Scopolamine-Induced Memory Impairment in the Morris Water Maze Test

**FIGURE 3:**
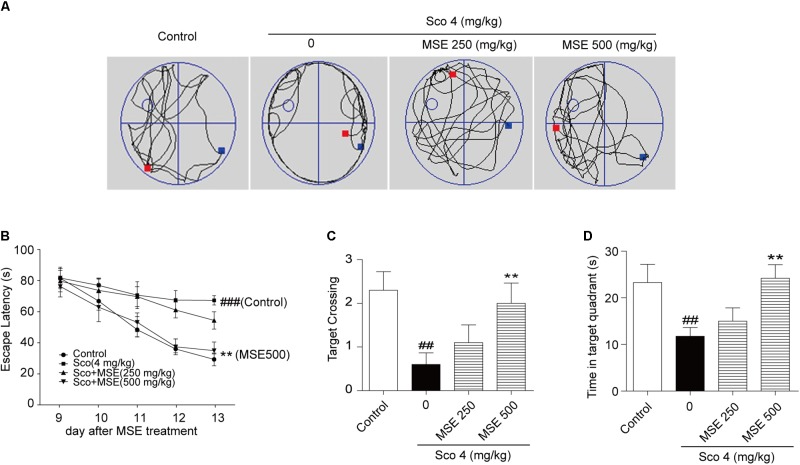
*Moringa oleifera* seed extract ameliorated scopolamine-induced spatial cognitive deficits on the Morris water maze (MWM) test. MSE (250 or 500 mg/kg) was administered to mice by oral gavage for 14 days, and memory impairment was induced by intraperitoneal injection of scopolamine (4 mg/kg) for 6 days. **(A)** Representative swimming traces on the spatial probe trial for each group. **(B)** Escape latency in the hidden platform trials. **(C,D)** The number of target platform crossings and time spent in the target quadrant during spatial probe trials. Data are expressed as the means ± SEM. *N* = 12 for each group. ^##^*P* < 0.01, ^###^*P* < 0.001 vs. control group, ^∗∗^*P* < 0.01 vs. scopolamine+vehicle group.

To further detect the influence of MSE on scopolamine-induced cognitive impairment, we exposed animals to the water maze task after 14 days of MSE treatment. Spatial learning was evaluated by the time required to find the hidden platform (escape latency). **Figure 3A** illustrates swim paths of animals on the sixth day of the water maze test. Scopolamine-treated mice spent nearly the same amount of time in the different quadrants of the pool, and swimming traces were uniformly distributed among the four zones. However, the swimming traces of the control group and scopolamine-treated mice that also received 500 mg/kg MSE were concentrated in the target zone, where the platform had been set. On day 13, group comparisons revealed that mice in the scopolamine-induced group displayed a longer latency in finding the platform than did animals in the control group (**Figure 3B**). These results indicated that scopolamine resulted in a significant impairment of cognitive acquisition and confirmed the usefulness of this model in detecting MSE effects on memory ability. After treatment with 500 mg/kg MSE, escape latency was markedly reduced on day 5 (**Figure 3B**). These data showed that pretreatment with 500 mg/kg MSE effectively ameliorated spatial learning across the 5-day training period. There were no significant differences between the 250 mg/kg MSE-treated and scopolamine-impaired mice in escape latency (**Figure 3B**). The platform was removed in the spatial probe trial, and scopolamine-impaired mice crossed the platform position less frequently and spent less time searching in the target quadrant than did control mice (**Figures 3C,D**). Compared with the scopolamine-impaired group, the 500 mg/kg MSE group exhibited obviously increased time in the target quadrant and more frequent crossing of the platform position (**Figures 3C,D**). Therefore, MSE treatment ameliorates scopolamine-induced spatial memory impairments. We additionally measured the swimming speed of all mice and found no differences, suggesting that these animals have normal motor function (data not shown).

### Effects of MSE on ACh and AChE Levels and ChAT Protein Expression in the Hippocampi of Scopolamine-Impaired Mice

**FIGURE 4:**
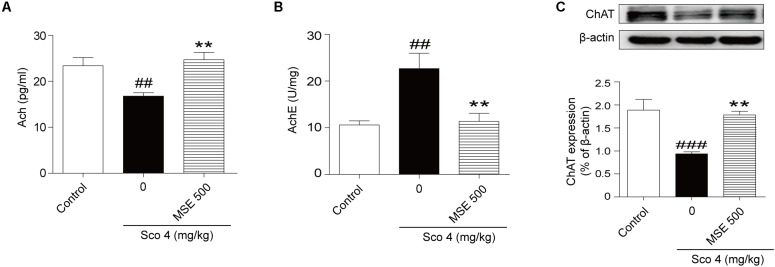
Effects of MSE (500 mg/kg) on acetylcholine (ACh) levels, acetylcholinesterase (AChE) activity and ChAT expression in the hippocampi of scopolamine-impaired mice. Mice were decapitated immediately after the MWM test, and the hippocampi were removed to determine ACh level, AChE activity, and ChAT expression. **(A,B)** Densitometry analysis of ACh and ChAT protein levels and **(C)** representative western blot gel in the hippocampus. Quantified results were normalized to β-actin expression. Data are presented as the means ± SEM (*n* = 12). ^##^*P* < 0.01, ^###^*P* < 0.001 vs. control group, ^∗∗^*P* < 0.01 vs. scopolamine +vehicle group.

The central cholinergic system is well known to play a major role in cognitive function, which is strongly modulated by the neurotransmitter ACh. Therefore, samples of hippocampus taken from mice were used for evaluation of cholinergic system reactivity after MWM test. Scopolamine produced a significant decrease of ACh content in the hippocampus (**Figure 4A**). Moreover, pretreatment with 500 mg/kg MSE significantly reversed the decrease in ACh levels induced by scopolamine (**Figure 4A**). Compared with the control group, the scopolamine-treated group showed significant enhancement of AChE activity in hippocampal tissue (**Figure 4B**), while pretreatment with 500 mg/kg MSE completely inhibited the hyperactivation of AChE induced by scopolamine (**Figure 4B**). ChAT, a key enzyme modulating ACh content in brain, is considered the definitive marker of central cholinergic function. The scopolamine-treated group showed significantly less ChAT level in the hippocampus than did the control group (**Figure 4C**). However, pretreatment with 500 mg/kg MSE significantly increased ChAT expression (**Figure 4C**).

### Effects of MSE on Neurogenesis and Synaptic Plasticity in the Hippocampus

**FIGURE 5:**
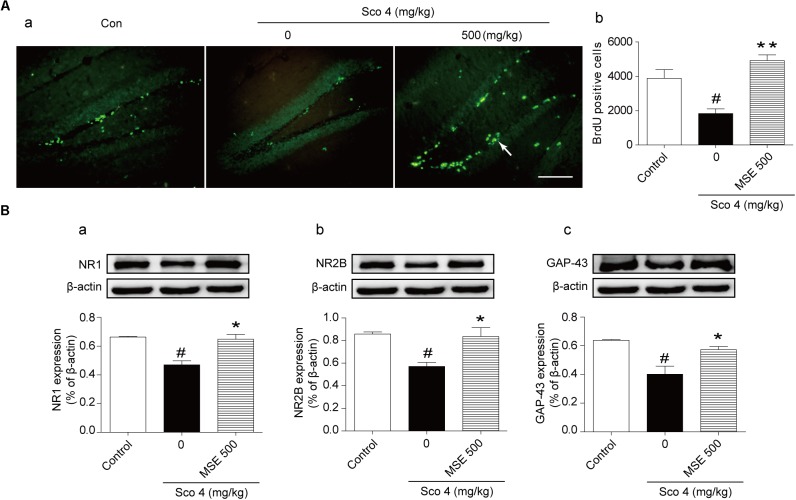
*Moringa oleifera* seed extract (500 mg/kg) promoted proliferation of neural precursor cells in the DG of the hippocampus. Mice were decapitated immediately after the MWM test, and hippocampal BrdU immunoreactive cells were examined using immunofluorescence. **(A-a)** Representative fluorescence images of BrdU immunoreactive cells (white arrow) on day 14 after MSE pretreatment. **(A-b)** Quantitative analysis of BrdU positive cells on day 14 after MSE pretreatment. **(B)** Western blots of NR1 **(B-a)**, NR2B **(B-b)**, GAP-43 **(B-c)** in the hippocampus on day 14 after MSE pretreatment. Quantified results were normalized to β-actin expression. The values are represented as the means ± SEM. *N* = 12 mice were used for immunohistochemistry, and *n* = 5 were used for western blot per group. ^#^*P* < 0.05 vs. control group, ^∗^*P* < 0.05, ^∗∗^*P* < 0.01 vs. scopolamine+vehicle group. Bar = 50 μm.

Scopolamine injection significantly suppressed the birth of new neural precursor cells in the hippocampal DG region, particularly in the SGZ, as evidenced by reduced BrdU staining (**Figure 5A-a**). These effects in the 500 mg/kg MSE pretreatment group were significantly reversed compared with those in the scopolamine-impaired group (**Figure 5A-b**). In the hippocampus, NR1, NR2B, and GAP-43 levels in scopolamine-treated mice were significantly lower than in the control group (**Figures 5B-a–c**). In contrast, the 500 mg/kg MSE group had significantly higher NR1, NR2B and GAP-43 protein levels in the hippocampus than did the scopolamine group (**Figures 5B-a–c**).

### Effects of MSE on Akt, ERK1/2, and CREB Signaling Pathways in Scopolamine-Impaired Mice

**FIGURE 6:**
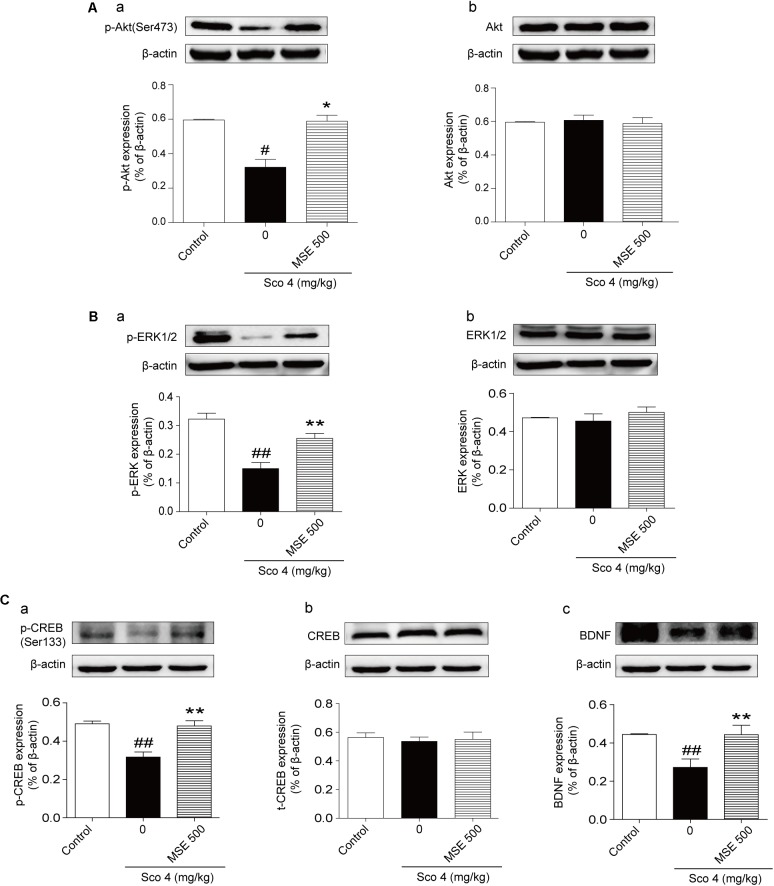
*Moringa oleifera* seed extract (500 mg/kg) treatment increased the Akt, ERK1/2 and CREB signaling pathways. Western blots of phosphor-Akt **(A-a)**, total Akt **(A-b)**, phosphor-ERK1/2 **(B-a)** and total ERK1/2 **(B-b)**, phosphor-CREB **(C-a)**, total CREB **(C-b)**, and BDNF **(C-c)** in the hippocampus 14 days after MSE pretreatment. Quantified results were normalized to β-actin expression. The values are represented as the means ± SEM. *N* = 5 for each group. ^#^*P* < 0.05, ^##^*P <* 0.01 vs. control group, ^∗^*P* < 0.05, ^∗∗^*P* < 0.01 vs. scopolamine+vehicle group.

Compared with the control group, the scopolamine-treated group exhibited significant down-regulation of the phosphorylation levels of Akt at Ser473 and ERK1/2 at Thr202/Thr204 (**Figures 6A-a,B-a**). Compared with the scopolamine-treated group, 500 mg/kg MSE treatment significantly ameliorated these effects (**Figures 6A-a,B-a**). We did not observe a significant difference in total Akt or ERK1/2 levels among the groups (**Figures 6A-b,B-b**). Compared with control treatment, scopolamine treatment produced a robust decrease in the phosphorylation level of CREB (Ser133) and expression of BDNF in the hippocampus (**Figures 6C-a,C-c**). In contrast, compared with scopolamine treatment, pretreatment with 500 mg/kg MSE markedly increased phosphor-CREB (Ser133) and BDNF level (**Figures 6C-a,C-c**). Total CREB level did not differ between the groups (**Figure 6C-b**).

## Discussion

In the present study, we used scopolamine to investigate the effects of MSE on cognitive impairment. Our results demonstrated that MSE is able to protect mice from scopolamine-induced learning and memory dysfunction as assessed by the passive avoidance and MWM tests. MSE pretreatment significantly enhanced cholinergic system reactivity and neurogenesis in the hippocampus. Additionally, the levels of phosphorylated Akt, ERK1/2, and CREB in the hippocampus were significantly decreased by scopolamine, and these decreases were prevented by MSE treatment.

The effect of MSE on cognitive function in animal models of scopolamine-induced impairment was detected using the passive avoidance and MWM tests. These two behavioral tests are both hippocampus-dependent tasks. The passive avoidance task is a fear-motivated test to assess hippocampus-dependent associative memory function in rodents (Izquierdo et al., 2006). The MWM test is commonly used to assess hippocampus-dependent spatial memory (Vorhees and Williams, 2006). Therefore, the effects of MSE on associative and spatial learning and memory functions were evaluated following scopolamine-induced impairment. We performed a pilot dose-response experiment with MSE (250 or 500 mg/kg) and found that 500 mg/kg was effective in improving scopolamine-induced amnesic effects, including memory deficits on the passive avoidance and MWM tests. These data suggest that MSE ameliorates scopolamine-induced impairments on different types of memory tests. Therefore, the present results support the utility of MSE in models of memory impairment, such as the associative and spatial learning and memory deficits.

The cholinergic system plays a vital role in learning and memory. Inhibition of cholinergic transmission may be responsible, at least in part, for cognitive impairments in models of scopolamine-induced amnesia (Hirokawa et al., 1996). In the present study, we used scopolamine to detect the effects of MSE in the cholinergic system. ACh is an essential neurotransmitter related to learning and memory processes, and strategies to enhance ACh level can improve cognitive function (Pepeu and Giovannini, 2004). ACh is unique among the classical neurotransmitters because its synaptic action is terminated by ACh hydrolysis by AChE (Ballard et al., 2005). However, excessive AChE activity results in constant ACh deficiency and cognitive deficits (Deak et al., 2016). Therefore, inhibition of AChE activity serves as a therapeutic target for the treatment of senile dementia, AD and Parkinson’s disease. ChAT is responsible for ACh biosynthesis and is essential for cholinergic neurotransmission in the CNS (Giacobini, 2002). The expression and activation of AChE and ChAT regulate the dynamic level of ACh in cholinergic synapses in the AD brain (Giacobini, 2002). We found that repeated scopolamine administration caused a reduction in ACh levels and ChAT expression as well as an increase in AChE activity in the hippocampus. However, MSE pretreatment significantly elevated ACh levels and ChAT expression and inhibit AChE activity in hippocampal tissues of the scopolamine-treated mice. These findings indicate that the anti-amnesic effects of MSE may be due to improvement of cholinergic neurotransmission system.

Adult hippocampal neurogenesis plays a vital role in hippocampal cognitive function (Vivar, 2015). BrdU is an analog of thymidine and can be incorporated into the DNA of cells during the S phase. Thus, it has been used to check cell proliferation (Doeppner et al., 2009). In the present study, we found that MSE treatment for 2 weeks by oral administration clearly increased the quantity of BrdU^+^ cells in the DG of the hippocampus, suggesting that MSE promoted basal neurogenesis in scopolamine-impaired mice. Learning and memory are not only closely related to cholinergic neurotransmission but also related to glutamatergic neurotransmission, which involves the *N*-methyl-D-aspartate (NMDA) receptor in the CNS (Whitlock et al., 2006). The effect of NMDA receptor signaling on learning and memory in the CNS is well established (Rao and Finkbeiner, 2007). Enhanced activation of NMDA receptor signaling results in facilitation of learning and memory in various behavioral tests (Shimizu et al., 2000). To investigate whether NMDA receptor signaling is involved in the effects of MSE on cognitive function, we evaluated the expression of NR1 and NR2B using Western blots. Our results showed that MSE treatment significantly increased the expression of both NR1 and NR2B, suggesting that NMDA receptor signaling may be implicated in the beneficial effects of MSE on cognitive dysfunction. In particular, the NMDA receptor plays a vital role in synaptic plasticity, which has been implicated in learning and memory (Nakanishi, 1992). To better understand the effects of MSE on cognitive deficits in scopolamine-treated mice, we assessed the effects of MSE pretreatment on synaptic plasticity. GAP-43 is an intracellular growth protein that plays a vital role in the regulation of growth cone guidance, synaptic plasticity and neurite outgrowth (Aigner et al., 1995). Our results demonstrate a significant decrease in GAP-43 levels in hippocampal tissue after scopolamine treatment. However, MSE pretreatment significantly increased expression of GAP-43. Thus, the effect of MSE on cognitive functional recovery may be attributable to MSE-induced neurogenesis and synaptic plasticity in the hippocampus.

The activation of mAChRs by ACh is known to induce the elevation of intracellular calcium levels, phosphoinositol turnover and activation of several kinases such as protein kinase A (PKA), protein kinase B (Akt), and ERK1/2(Bonner et al., 1987; Felder, 1995; Rosenblum et al., 2000). Akt activation leads to expression of learning-related proteins. Akt has been reported to play a vital role in synaptic plasticity. Inhibition of Akt activation causes impairments in fear-related learning, passive avoidance learning and spatial learning (Barros et al., 2001). ERK1/2, another signaling pathway, is involved in learning and memory (Giovannini, 2006). Because Akt and ERK1/2 are related to learning and memory processes, agents that affect activation of Akt and ERK1/2 may have potential benefits for the treatment of AD. To evaluate molecular mechanisms of MSE on improving cognitive functions, we measured phosphorylation levels of Akt and ERK1/2 in the hippocampus after MSE pretreatment. Our data showed that MSE pretreatment reversed the inhibition of Akt and ERK1/2 activation induced by scopolamine injection, suggesting that the cognitive improvements of MSE may be related to mitigation of scopolamine-induced Akt and ERK1/2 inactivation. CREB is a downstream nuclear factor of PKA and is essential for synaptic plasticity and memory in the CNS. Previous studies have shown that activation of CREB ameliorates cognitive impairment via the cholinergic system (Kotani et al., 2006). BDNF is known to improve learning function and neurogenesis via activation of CREB signaling (Bimonte-Nelson et al., 2008). To further evaluate the molecular mechanisms of MSE on learning and memory function, the effects of MSE on CREB activation and BDNF expression in the hippocampus were assessed. In accordance with previous studies (Kotani et al., 2008; Joh et al., 2012; Kim et al., 2013), our results showed that the phosphorylation level of CREB and the expression of BDNF in the hippocampus were inhibited by treatment with scopolamine. However, these changes were reversed by MSE pretreatment. Taken together, our results suggest that the beneficial cognitive effects of MSE may be related to activation of the Akt, ERK1/2, and CREB signaling pathways.

A previous report indicated that MSE extract primarily comprises either hydrocarbons or long chain polyunsaturated fatty acids and their derivatives, including polyunsaturated fatty acids (PUFAs) (Al-Asmari et al., 2015). Supplementation of PUFAs increases synaptic plasticity in the hippocampus and improves cognitive function (Das, 2008; Bazinet and Laye, 2014). The ability of MSE to improve learning and memory may be associated with PUFAs. However, determining which ingredients in MSE promote learning and memory function requires further experimentation.

## Conclusion

**FIGURE 7:**
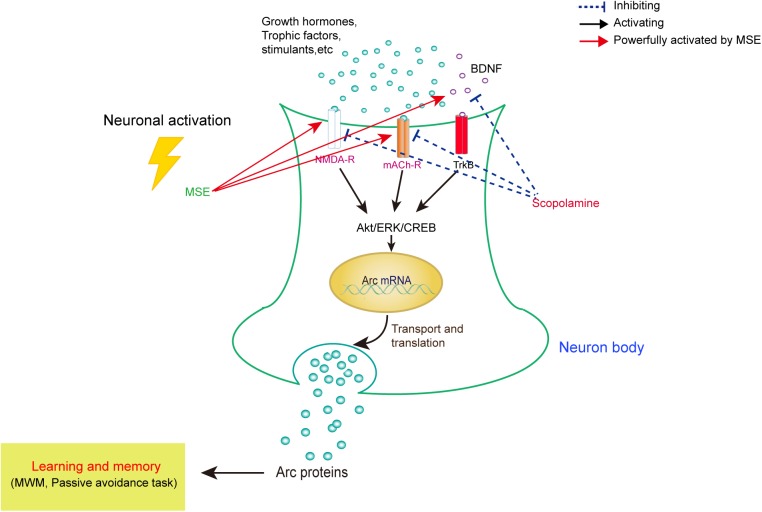
Model for correlating the modulation of learning and memory with MSE-mediated changes in the scopolamine-induced amnesic mouse brain. In amnesic mice, MSE may promote cholinergic system reactivity through activation of the Akt, ERK1/2, and CREB signaling pathways.

As summarized in **Figure 7**, our data demonstrate that MSE has an anti-amnesic effect, which could be mediated by cholinergic activity, hippocampal neurogenesis and the Akt/ERK1/2/CREB signaling pathways. These findings suggest that the *M. oleifera* seed may be a promising treatment for patients with neurodegenerative disorders. Further studies should aim to confirm these neuroprotective effects and their corresponding mechanisms using active compounds of *M. oleifera* seed.

## Author Contributions

L-cY and XJ conceived and designed the experiments. JZ, W-sY, YL, LP, K-yZ, YW, and TR performed the experiments. YuZ and YunZ analyzed the data. JZ, W-sY, and D-qS wrote the paper. All authors reviewed and gave final approval.

## Conflict of Interest Statement

The authors declare that the research was conducted in the absence of any commercial or financial relationships that could be construed as a potential conflict of interest.

## Supplementary Material

FIGURE S1 Experimental design and schedule. In experiment 1, mice were orally pretreated with *Moringa oleifera* seed extract (MSE) (250 or 500 mg/kg) for 7 days, and scopolamine (4 mg/kg) was injected intraperitoneally 30 min before the step-down test on day 8. Twenty-four hours after the acquisition trial, a retention trial was conducted for 300 s. In experiment 2, mice were orally pretreated with MSE (250 or 500 mg/kg) for 7 days, and scopolamine (4 mg/kg) was injected intraperitoneally 30 min before the step-through test on day 8. Twenty-four hours after the acquisition trial, a retention trial was conducted for 300 s. In experiment 3, MSE (250 or 500 mg/kg) was administered to mice by oral gavage for 14 days (days 1–14), and memory impairment was induced by intraperitoneal injection of scopolamine (4 mg/kg) for 6 days (days 9–14). BrdU (50 mg/kg, *i.p*.) was given twice daily during days 1–7. BrdU immunohistochemistry was performed on day 14 after MSE pretreatment.
