# Supplementary figures and images for: Moringa oleifera Seed Extract Alleviates Scopolamine-Induced Learning and Memory Impairment in Mice
Source: Front Pharmacol. 2018 Apr 24;9:389. doi: 10.3389/fphar.2018.00389 (PMC5928465; doi:10.3389/fphar.2018.00389)

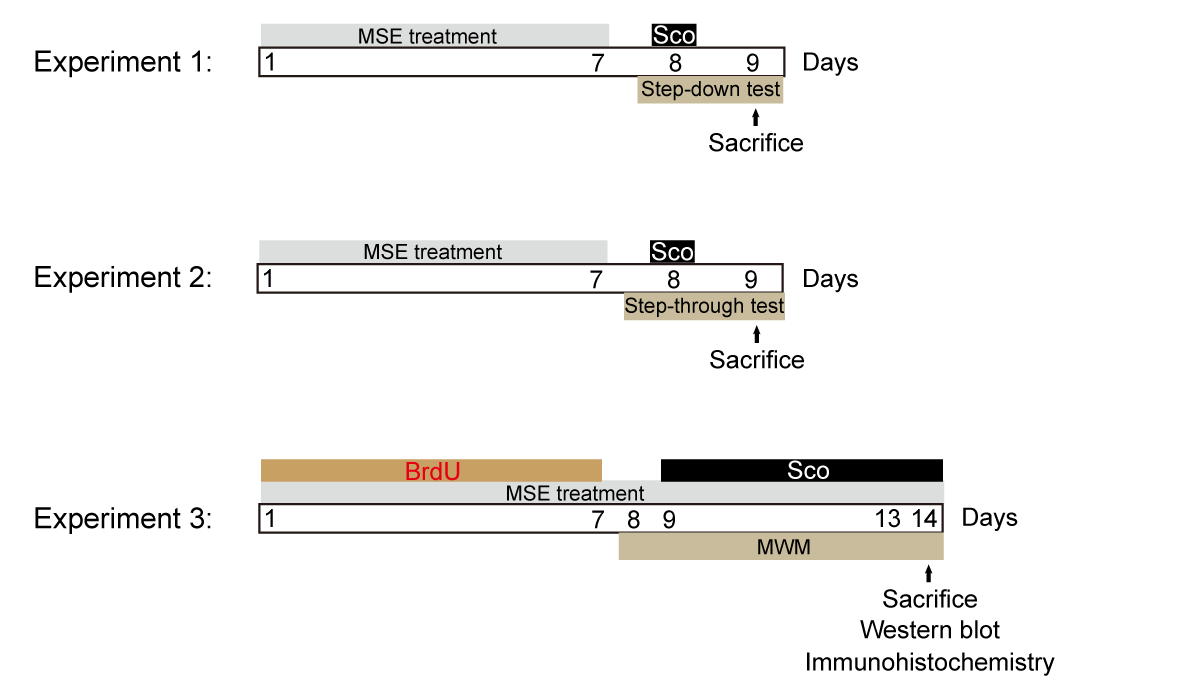

Supplement: FIGURE S1 — Experimental design and schedule. In experiment 1, mice were orally pretreated with Moringa oleifera seed extract (MSE) (250 or 500 mg/kg) for 7 days, and scopolamine (4 mg/kg) was injected intraperitoneally 30 min before the step-down test on day 8. Twenty-four hours after the acquisition trial, a retention trial was conducted for 300 s. In experiment 2, mice were orally pretreated with MSE (250 or 500 mg/kg) for 7 days, and scopolamine (4 mg/kg) was injected intraperitoneally 30 min before the step-through test on day 8. Twenty-four hours after the acquisition trial, a retention trial was conducted for 300 s. In experiment 3, MSE (250 or 500 mg/kg) was administered to mice by oral gavage for 14 days (days 1–14), and memory impairment was induced by intraperitoneal injection of scopolamine (4 mg/kg) for 6 days (days 9–14). BrdU (50 mg/kg, i.p.) was given twice daily during days 1–7. BrdU immunohistochemistry was performed on day 14 after MSE pretreatment. [file Image_1.TIF]
